# Interplay between DNA sequence and negative superhelicity drives R-loop structures

**DOI:** 10.1073/pnas.1819476116

**Published:** 2019-03-08

**Authors:** Robert Stolz, Shaheen Sulthana, Stella R. Hartono, Maika Malig, Craig J. Benham, Frederic Chedin

**Affiliations:** ^a^Department of Molecular and Cellular Biology, University of California, Davis, CA 95616;; ^b^Integrative Genetics and Genomics Graduate Group, University of California, Davis, CA 95616;; ^c^Department of Mathematics, University of California, Davis, CA 95616;; ^d^Department of Biomedical Engineering, University of California, Davis, CA 95616;; ^e^Genome Center, University of California, Davis, CA 95616

**Keywords:** R-loop, DNA topology, transcription, modeling

## Abstract

R-loops are abundant three-stranded nucleic-acid structures that form *in cis* during transcription. Experimental evidence suggests that R-loop formation is affected by DNA sequence and topology. However, the exact manner by which these factors interact to determine R-loop susceptibility is unclear. To investigate this, we developed a statistical mechanical equilibrium model of R-loop formation in superhelical DNA. In this model, the energy involved in forming an R-loop includes four terms—junctional and base-pairing energies and energies associated with superhelicity and with the torsional winding of the displaced DNA single strand around the RNA:DNA hybrid. This model shows that the significant energy barrier imposed by the formation of junctions can be overcome in two ways. First, base-pairing energy can favor RNA:DNA over DNA:DNA duplexes in favorable sequences. Second, R-loops, by absorbing negative superhelicity, partially or fully relax the rest of the DNA domain, thereby returning it to a lower energy state. In vitro transcription assays confirmed that R-loops cause plasmid relaxation and that negative superhelicity is required for R-loops to form, even in a favorable region. Single-molecule R-loop footprinting following in vitro transcription showed a strong agreement between theoretical predictions and experimental mapping of stable R-loop positions and further revealed the impact of DNA topology on the R-loop distribution landscape. Our results clarify the interplay between base sequence and DNA superhelicity in controlling R-loop stability. They also reveal R-loops as powerful and reversible topology sinks that cells may use to nonenzymatically relieve superhelical stress during transcription.

## Significance

Three-stranded R-loop structures form during transcription when the nascent RNA transcript rehybridizes to the template DNA strand. This creates an RNA:DNA hybrid and forces the nontemplate DNA strand into a single-stranded, looped-out state. R-loops form universally over conserved hotspot regions. To date, the physicochemical bases underlying R-loop formation remain unclear. Using a “first-principle” mathematical approach backed by experimental validation, we elucidated the relative contributions of DNA sequence and DNA topology to R-loop formation. Our work provides a quantitative assessment of the energies underlying R-loop formation and of their interplay. It further reveals these structures as important regulators of the DNA topological state.

R-loops are three-stranded nucleic-acid structures that form when the nascent RNA transcript hybridizes with the template DNA strand, leaving the nontemplate strand unpaired (1, 2). The sensitivity of this unpaired strand to nondenaturing bisulfite treatment allows the occurrence and locations of R-loops to be determined on single DNA molecules (3). When applied to chromosomes extracted from mammalian cells, this approach has shown that R-loops can be long, often spanning several hundred base pairs, and reaching maximal lengths of up to 2 kilobases (3–5). Genomic profiling studies using the S9.6 anti-RNA:DNA hybrid antibody have established that R-loops are prevalent, covering 3–5% of the genomic space in organisms from yeasts (6–9) to plants (10) and mammals (4, 11–14). Global R-loop maps show that R-loop formation does not result from random trapping of the nascent RNA. Instead, R-loops are observed over tens of thousands of broadly conserved hotspots that are enriched at gene ends (10, 11, 15, 16).

Although R-loops are abundant and biologically relevant (17), relatively little is known about the factors that determine their occurrence. They form efficiently in G-rich transcripts (18, 19), owing to the high thermodynamic stability of riboG:deoxyC RNA:DNA hybrids (20–23). GC-rich DNA sequences that are also GC-skewed (i.e., show strand asymmetry in the distribution of G and C bases) are prone to R-loop formation both at endogenous genomic loci (3–5) and upon in vitro transcription (3, 4, 18, 19). G clusters, in particular, were shown to be strong initiation points for RNA strand invasion, leading to R-loop formation upon extension (24, 25).

Experimental evidence suggests that R-loop formation is also affected by DNA topology. In both *Escherichia coli* and yeast, hypernegative supercoiling resulting from Topo1 inactivation leads to an increased frequency of R-loops in highly transcribed ribosomal regions (26–29). In human cells, transient knockdown of Top1 also leads to R-loop gains at rDNA sequences and over long highly transcribed genes where supercoil dissipation was constrained (30). The negative superhelicity commonly found in bacterial DNA or transiently imposed behind the RNA polymerase in eukaryotes (31–33) is thought to favor R-loop formation. However, the exact manner by which DNA topology interacts with sequence-based physicochemical properties to determine R-loop susceptibility has not been examined to date.

Here, we present an equilibrium statistical mechanical model that analyzes R-loop susceptibilities in superhelical DNA sequences. Our approach is similar to that used to treat other superhelically driven transitions, such as strand separation and B/Z transitions (34–39). We have used this model to describe the interplay between DNA topology and base sequence in stabilizing R-loops. In vitro transcription assays and single-molecule R-loop footprinting establish that negative superhelicity and DNA sequence together regulate R-loop stability and determine the R-loop distribution landscape. Our model suggests that these effects are due to the ability of R-loops to absorb negative superhelicity, thereby returning the DNA fiber to a more energetically favorable partially or fully relaxed state. R-loops with more favorable base-pairing properties require less energy return from DNA relaxation, while R-loop formation over less-favorable regions requires significantly more negative superhelicity for their formation and stability.

## Results

### An Energy-Based Model for R-Loops.

Equilibrium statistical mechanics assesses the frequencies with which alternate molecular conformations occur in a population at equilibrium, based on their energetics (40). The relative frequency of a state *s* varies with the free energy *G*(*s*) of that state, according to its Boltzmann factor *e^-G^*^(^*^s^*^)/^*^RT^*, where *R* is the gas constant and *T* the absolute temperature.

Here, we have applied this approach to R-loops. We considered a duplex DNA sequence containing *N* base pairs that is superhelically constrained with a specified linking difference α. In principle, an R-loop of any length can occur at any position within this topological domain, provided the cRNA exists. Once a free energy is assigned to each state, including the state with no R-loop, equilibrium values of any parameters of interest (e.g., R-loop lengths, probabilities, or energies) can be computed as described in *SI Appendix*. This model is not concerned with how R-loops dynamically arise or dissipate, but only with their relative stabilities at the given level of superhelicity.

**Fig. 1.**
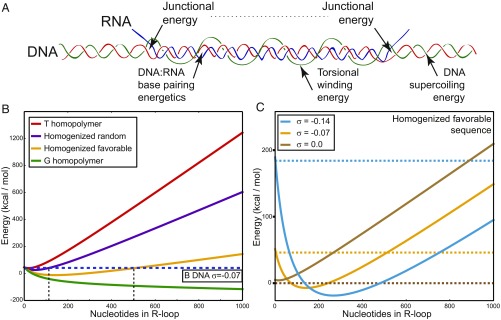
(*A*) Schematic representation of an R-loop showing the four sources of energy used in the energy model. Here, the unpaired single strand of the R-loop wraps around the RNA:DNA hybrid in a left-handed helix. (*B*) The energies of R-loops formed with various homogenized DNA sequences are graphed as a function of R-loop length. Each sequence is color coded as indicated. The energy of B DNA under these conditions (σ = −0.07) is shown as a horizontal dashed line. The vertical dashed lines highlight the maximal predicted length of R-loops formed on a random and a favorable sequence, respectively. (*C*) The energies of R-loops formed on a favorable DNA sequence as a function of R-loop length are graphed at three levels of supercoiling, as indicated. The energy of B DNA at each supercoiling level is indicated by a horizontal dashed line of the same color.

Suppose a specific state has an R-loop that contains *m* DNA:RNA hybrid base pairs, starting at position *n* + 1 and ending at position *n* + *m*. In our model, the energy needed to produce this state has four terms (Fig. 1*A*). First, the R-loop disrupts *m* DNA:DNA base pairs and forms a DNA:RNA hybrid with the same sequence. The base-pair energy required for this is *B* = *b*_hybrid_ − *b*_duplex_. Values of *B* have been measured for all nearest-neighbor pairs (21). Seven of the 16 possible neighbor pairs have negative *B* values, meaning that for them, the hybrid is more stable than the DNA duplex. Second, every R-loop has two junctions, where it joins back to the DNA duplex. This pair of junctions requires substantial energy to form. While the value of this junction energy *a* has not been measured specifically for R-loops, it is known for both B/Z transitions and local strand separation that 10 < *a* < 11 (kcal/mol) (37, 41–43). Here, we used *a* = 10.5 kcal/mol. This highly unfavorable junction energy must be overcome before an R-loop becomes stabilized. Third, the presence of an R-loop alters the manner in which superhelicity is partitioned in two ways. Dissociation of the DNA duplex changes the unstressed twist from the *mA* characteristic of the duplex to zero (here, *A* = 1/10.5 turns per bp is the average helicity of the B form). This untwisting absorbs negative superhelicity, leaving α + *mA* turns available to stress the rest of the domain (note that the total superhelicity α remains fixed in all states, but that it gets partitioned differently when an R-loop is present). Finally, because single-stranded DNA is flexible, the unpaired nontemplate strand can wind around the DNA:RNA hybrid with helicity τ (radians per base). This leaves a residual superhelicity α_r_ = α + *mA* − *m*τ/2π to superhelically deform the domain. The free energies associated with these last two effects are both quadratic, with coefficients *K* for superhelicity and torsional stiffness *C* for the winding of the unpaired strand. If we let these two effects equilibrate, then the total free energy *G*(*s*) associated to this state is:

$$G\left(s\right)=\{\begin{array}{c} \frac{1}{2}K\alpha^{2},m=0\\ a+\sum_{i=1}^{m}B\left(n+i\right)+\frac{2\pi^{2}CK}{4\pi^{2}C+Km}{\left(\alpha +mA\right)}^{2},m>0 \end{array}.$$ [1]

(The state with no R-loop corresponds to *m* = 0.) The values of all of the energy parameters used here were taken directly from the literature; none were optimized to fit data. The full model, including the derivation of this equation and full set of parameter values used, is presented in *SI Appendix*.

We developed an algorithm to perform calculations based on this model for DNA domains having any base sequence and any level of superhelicity. The topological domain may be either closed circular like a plasmid or a portion of a superhelically constrained loop. The entire domain may be regarded as susceptible to R-looping or only a portion of it. The C++ implementation of this algorithm, called R-looper, enumerates R-loop states, calculates their energies from Eq. **1**, and assigns a Boltzmann factor to each. This information is used to calculate the equilibrium probability of each state as a function of superhelicity and DNA sequence. Other equilibrium properties can then be calculated at each position, including the probability of R-loop formation and average values of R-loop lengths and free energies. In this implementation, we only considered states having at most one R-loop because the high value of the junction energy *a* strongly disfavors states with multiple R-loops.

### R-Loop Equilibria Are Sensitive to both DNA Sequence and Topology.

To assess the role of sequence in R-loop stability, we ran our model on various uniform sequences at superhelical density σ = −0.07 (σ = α/*NA*) and compared the free energy of R-loops of each length to the free energy of the B-form state having no R-loop (Fig. 1*B*). We used four sequences spanning the range from least to most favorable. The most unfavorable sequence for R-loops is a T homopolymer, for which the base pair energy is *B* = +0.8 kcal/mol. In this case, R-loops of all lengths were predicted to be energetically disfavored relative to the B-form state. Next, we used a homogenized random sequence, for which the base-pair energy was set at the constant value of *B* = +0.23 kcal/mol, corresponding to the average energy of all 16 nearest-neighbor pairs. In this sequence and level of superhelicity, short R-loops of lengths up to ∼100 bp were energetically favored over the B form. By contrast, R-loops of lengths up to 500 bp were energetically favored for a homogenized favorable sequence (*B* = −0.15 kcal/mol, corresponding to the average energy of all seven favorable nearest-neighbor pairs). R-loops were favored over B DNA at all lengths for the most favorable, G homopolymer sequence (*B* = −0.36 kcal/mol).

Our model also predicted that R-loops are highly sensitive to DNA superhelicity. Fig. 1*C* plots the energy of R-loops for the homogenized favorable sequence at three levels of negative superhelicity. In each case, the energy of the state with no R-loop (B DNA) is represented by the horizontal dotted line of the same color. Although R-loops of length up to 500 bp are favored in this sequence at σ = −0.07, the model predicted that no R-loop states are energetically favored over the B form when the same sequence is relaxed (σ = 0). When a highly negative superhelical density was imposed (σ = −0.14), R-loops were predicted to be highly favored, with lengths up to 1,400 bp. Thus, our model predicts that both DNA sequence and topology strongly influence R-loop formation.

**Fig. 2.**
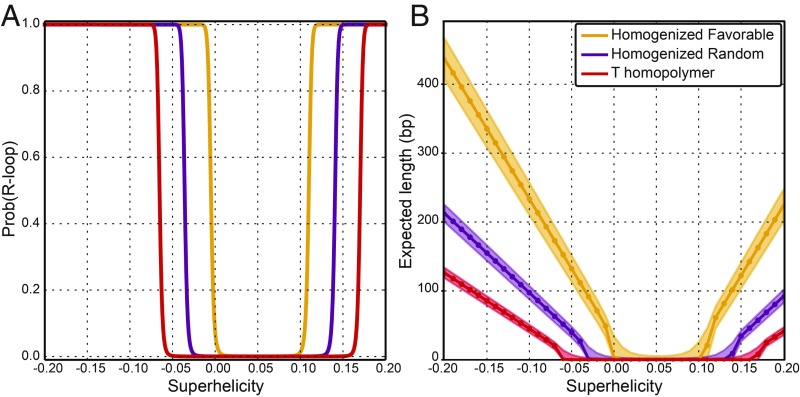
(*A*) The probability that an R-loop occurs on three distinct homogenized DNA sequences is graphed as a function of the DNA template superhelix density σ. (*B*) The ensemble average lengths expected for R-loops formed on three distinct DNA sequences are graphed as a function of σ. The shaded areas represent one standard deviation (SD) from the mean.

To analyze the relationship between DNA topology and R-loops further, we calculated the equilibrium probability of an R-loop occurring as a function of superhelicity. As shown in Fig. 2*A*, our model predicted that even the most unfavorable T homopolymer sequence can be driven into an R-loop by sufficient negative superhelicity, while more energetically favorable sequences required less superhelicity to form R-loops. For favorable sequences, even slight amounts of negative superhelicity were sufficient to stabilize this transition. Interestingly, the model also predicted that high-positive superhelicity can drive R-loop formation. For instance, R-loops were predicted to occur above σ = +0.1, a physiologically attainable value, in the favorable sequence (*B* = −0.15 kcal/mol). This behavior is a consequence of the high flexibility of single-stranded DNA. Wrapping of the unpaired, nontemplate strand around the DNA:RNA hybrid can be either left-handed, absorbing negative supercoils, or right-handed, absorbing positive supercoils. However, the latter effect can only happen if this wrapping has a helicity greater than that of the B-form helix, which is only feasible at high-positive superhelicities. Indeed, R-loops were predicted at moderate levels of negative superhelicity, but only at much higher levels of positive superhelicity (*SI Appendix*, Fig. S1).

Our model also predicted that increasing levels of negative DNA superhelicity stabilize R-loops of increasing size (Fig. 2*B*). For all tested sequences, R-loops started growing in length as soon as they became favorable. However, for energetically less-favorable sequences, R-loops required more superhelicity to initiate and grew more slowly. In the homogenized favorable sequence at σ = −0.07, the predicted ensemble average R-loop length was 180 bp. By contrast, the unfavorable T homopolymer sequence was only beginning to experience short R-loops (average length of 25 bp) at this superhelix density. While R-loop formation became possible at high-positive superhelicity, the average lengths of the structures remained much shorter than those predicted for the corresponding level of negative superhelicity (Fig. 2*B*). Thus, increasing superhelicity was predicted to increase both the probability and length of R-loop structures, with other factors being fixed.

### Relaxation of DNA Superhelicity by R-Loop Formation.

According to our model, R-loops are favored at equilibrium, primarily because they absorb negative superhelicity through base unpairing and strand twisting, thereby allowing the rest of the domain to relax. R-loops become favored at superhelicities where the base-pairing energies *B* and the superhelical relaxation together are enough to overcome the unfavorable junction free energy *a.* Beyond that point, longer R-loops occur at higher negative superhelicity because they provide more relaxation.

**Fig. 3.**
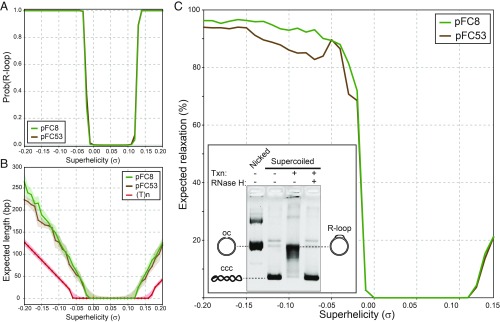
(*A*) The probability of R-loop formation is plotted as a function of superhelix density σ for the pFC8 (green) and pFC53 (brown) plasmids, as calculated by using R-looper. (*B*) Expected R-loop lengths are plotted for each plasmid as a function of σ. The shaded area around each curve delineates ±1 SD around the mean, shown as the central line. Since sufficient superhelicity will drive any sequence to form an R-loop, we included the expected length for the T homopolymer as a “negative” control. (*C*) This graph shows the percent relaxation of plasmid superhelicity expected from R-loop formation as a function of the initial template superhelix density σ for both pFC53 and pFC8. The expected relaxation is calculated as the fraction of superhelicity left after R-loop formation, (σ − σ_r_)/σ. *C*, *Inset* shows the results of an in vitro transcription experiment in which negatively supercoiled pFC8 was transcribed to generate R-loops and treated with RNase H or not. R-loop formation was accompanied by a strong topological shift up to, or close to, the position of the relaxed, nicked substrate (open circle; oc). R-loop resolution by RNase H caused a full return of R-looped plasmids to the covalently closed circular (ccc) negatively supercoiled form.

To test these predictions, we applied the equilibrium model to two plasmids, pFC53 and pFC8. Each of these plasmids carries a portion of a GC-skewed CpG island region that forms R-loops in vivo and in vitro (4). pFC8 carries a 950-bp portion of the human *SNRPN* CpG island, while pFC53 carries a 1.4-kb portion of the murine *Airn* CpG island; both regions were cloned into the same backbone. We first examined how the probability of R-loop formation in these plasmids varied with superhelicity. Both plasmids were predicted to start forming R-loops at slightly negative superhelicities and to be refractory to R-loops when relaxed (Fig. 3*A*). Likewise, our model also predicted that expected R-loop lengths would increase with increasing superhelicity (Fig. 3*B*). We next calculated the fraction of superhelicity absorbed by R-loops as a function of initial template supercoiling. As superhelicity decreased just below zero, R-loops started occurring, and plasmid relaxation rapidly increased (Fig. 3*C*). Remarkably, up to 80–95% of the initial template superhelicity was absorbed at all values beyond σ = −0.04. This level of fractional relaxation was maintained, even at high levels of negative superhelicity, because progressively longer R-loops were predicted to form, with the average lengths of R-loops increasing approximately linearly with |σ| (Fig. 3*B*). This analysis suggests that R-loop formation significantly relaxes the superhelical stress on a susceptible plasmid. These findings are entirely consistent with the well-known behavior of R-loops in in vitro transcription assays, where R-loop formation is measured by the gel retardation resulting from the topological relaxation of the negatively supercoiled substrate (Fig. 3 *C*, *Inset*) (3, 4, 18, 44).

### Negative Supercoiling Facilitates R-Loop Formation in Vitro.

We used in vitro transcription assays on the pFC53 plasmid to test our prediction that superhelicity plays a major role in regulating R-loop formation. Negatively supercoiled plasmid substrates were purified, and their topology was enzymatically manipulated to form highly negatively supercoiled, relaxed, nicked, and linear molecules (*Materials and Methods*). The resulting changes in DNA topology were verified on 1D and 2D chloroquine gels (*SI Appendix*, Fig. S2). To capture R-loops formed during in vitro transcription on substrates devoid of any supercoiling (e.g., relaxed, nicked, and linear), the transcription products were further incubated with the S9.6 antibody, which binds with high affinity to RNA:DNA hybrids (45).

**Fig. 4.**
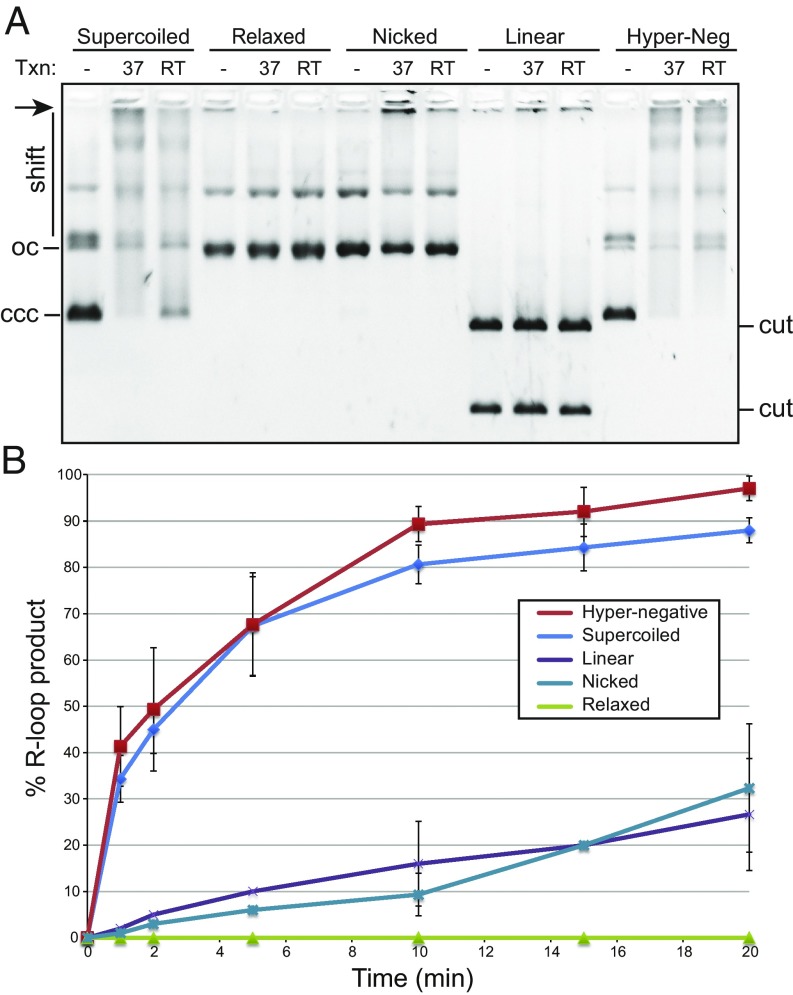
(*A*) In vitro transcription reactions were carried out on pFC53 at room temperature (RT) and 37 °C; posttranscription, the products were bound by the S9.6 antibody to supershift any RNA:DNA hybrid species and separated through an agarose gel. Shifted species tended to smear up and concentrate near the wells (arrow). R-loop formation is measured by the disappearance of the initial untranscribed substrate. The topological state of pFC53 was manipulated before transcription as indicated above, with linearization resulting in two cut bands. A representative gel obtained after a 20-min reaction is shown. R-loop formation is efficient for the supercoiled and even more so for the highly negatively supercoiled substrates. ccc, covalently closed circular; oc, open circle. (*B*) This graph plots the average R-loop yields upon transcription (at 37 °C) through pFC53 in various topological states, as a function of time. The curve shows the averages and SDs from three independent experiments.

As measured by the disappearance of the initial substrate, transcription through *Airn* on a negatively supercoiled template led to efficient R-loop formation at 37 °C and to a lesser extent at room temperature (RT; Fig. 4*A*). Consistent with model predictions, R-loop formation on highly negatively supercoiled plasmids was further enhanced, especially at RT. However, R-loop formation was sharply reduced on the Top1-relaxed, nicked, and linear templates. To better quantify the effect of DNA topology on R-loop formation efficiency, we repeated the in vitro transcription experiments over a 20-min time course at RT. Both negatively supercoiled plasmids efficiently formed R-loops, reaching 85–95% R-loop formation in 20 min (Fig. 4*B*). At every time point, structure formation was slightly more efficient for the highly negatively supercoiled substrates. Linear and nicked substrates were capable of R-loop formation, in agreement with prior data (46), but the initial reaction rates were 10- to 20-fold lower than for negatively supercoiled plasmids, and the final extent of the reaction was reduced threefold. The relaxed plasmid was refractory to R-loop formation, regardless of time. The observed variations in R-loop quantities were unlikely to be due to changes in transcription efficiency, as robust RNA synthesis was observed in every situation. These results strongly support the prediction that negative superhelicity is required for R-loop formation, even in favorable GC-skewed DNA sequences.

### The Equilibrium Model Accurately Predicts in Vitro R-Loop Locations.

**Fig. 5.**
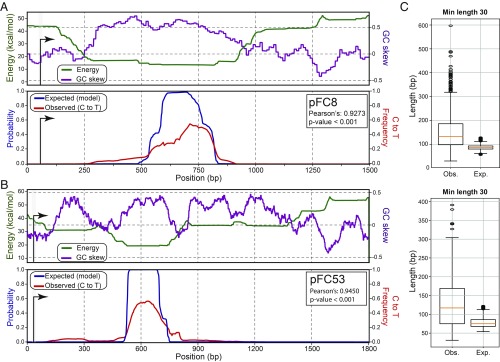
Comparison of model-derived expected signal and experimentally derived R-loops for negatively supercoiled pFC8 (*A*) and pFC53 (*B*) plasmids. *Upper* graphs in *A* and *B* display the model-derived ensemble average energy value for all predicted R-loops at any given base pair (green) and the corresponding GC skew for the region (purple). *Lower* graphs in *A* and *B* display ensemble average model-derived probability of R-loop formation at each base pair (blue) and the observed population average R-loop frequency along the region, as measured by the bisulfite-induced C-to-T conversion frequency measured over R-loop peaks (red). In both cases, transcription was conducted on supercoiled DNA molecules, and SMRF-seq was performed after linearization of the plasmid. (*C*) Boxplots of observed (Obs., left) and predicted (Exp., right) R-loop lengths for pFC8 (*Upper*) and pFC53 (*Lower*). The minimal lengths for experimentally derived R-loops was arbitrarily cut off at 30 bp.

We used our equilibrium model to predict the positions of R-loops on the pFC8 plasmid at superhelical density σ = −0.07. An energy-minimum valley was predicted in a 600-bp-long GC-skewed region located 200 bp downstream from the transcription start site (TSS) (Fig. 5*A*). The probability of R-loop formation rose sharply over the lowest energy portion of the valley, reaching a value of 1 over a 120 bp stretch.

To compare model predictions to experimentally generated R-loops, we subjected supercoiled pFC8 to in vitro transcription, then linearized the plasmid DNA and mapped the resulting R-loops using single-molecule R-loop footprinting (SMRF-seq). SMRF-seq exploits the sensitivity of unpaired cytosines, such as those on the looped out strand of R-loops, to nondenaturing bisulfite treatment (3). Bisulfite triggers deamination of unpaired cytosines to uracils, which, after PCR amplification, provide a permanent genetic tag for the presence of single-stranded DNA. Single-molecule sequencing of the amplicons provides a high-resolution, strand-specific readout of the positions of individual R-loops at high coverage (*SI Appendix*, Fig. S3*A*). SMRF-seq is entirely independent of the S9.6 antibody. Five highly reproducible biological replicates were performed (*SI Appendix*, Fig. S3*B*), and a total of 1,885 independent R-loop tracks, or footprints, were collected out of 3,912 sequenced molecules. These footprints were highly congruent and spread through a ∼300 bp area (*SI Appendix*, Fig. S3*C*). Over 94.3% of molecules carried single C-to-T conversion tracks, indicating that only one R-loop formed, consistent with model assumptions. As expected, R-loop footprints were highly specific to the nontemplate DNA strand. They also were transcription-dependent and largely RNase H-sensitive, as expected from genuine R-loops (*SI Appendix*, Fig. S3*D*). By plotting the average aggregate C-to-T conversion frequency over the amplicon, a population-average R-loop frequency signal was generated and compared with the theoretical model.

The vast majority of R-loop footprints were neatly confined within the predicted favorable region, with numerous footprints initiating and terminating at or near the predicted initiation and termination sites, respectively (Fig. 5*A* and *SI Appendix*, Fig. S3*E*). Pearson correlation analysis between the model-derived expected signal and the experimentally observed signal showed strong agreement, with a 0.93 correlation value. By contrast, GC skew correlated with the experimental signal with a lower Pearson correlation of 0.66.

The same approach was used for pFC53. As shown in Fig. 5*B*, our model again predicted a sharp peak of high R-loop probability centered over the lowest energy region located ∼400 bp downstream of the TSS. This region corresponds to one of several high-GC-skew subregions in this sequence. SMRF-seq generated a collection of 564 independent, strand-specific footprints from two independent replicates (*SI Appendix*, Figs. S3*D* and S4*A*). The footprints again defined a series of overlapping R-loop signals spread through an ∼300-bp region. A remarkable agreement between experimental and predicted data were observed, with the large majority of footprints mapping within the boundaries of the predicted region (Fig. 5*B* and *SI Appendix*, Fig. S4*B*). The Pearson correlation value between theoretical and experimental data for pFC53 was 0.94. By contrast, GC skew was less correlated (Pearson = 0.42) with the experimental signal.

For both plasmids, the observed length distributions of R-loop footprints were consistent with, but slightly higher than, their predicted lengths (Fig. 5*C*). This difference was due in part to the fact that our peak-calling threshold excludes short R-loops (<30 bp). Lowering this threshold (10 bp) led to a reduction of median observed lengths that matched more closely to the predicted lengths (*SI Appendix*, Fig. S4*C*). In both cases, nonetheless, a number of transcription-generated structures were significantly longer than predicted. This could occur because our model does not include the superhelicity that is transiently generated by transcription (30), thus underestimating the superhelical density. Alternatively, this could result from a dynamic effect during transcription that is not considered in our model.

### Superhelicity Is Essential for R-Loop Stability over Suboptimal Regions.

**Fig. 6.**
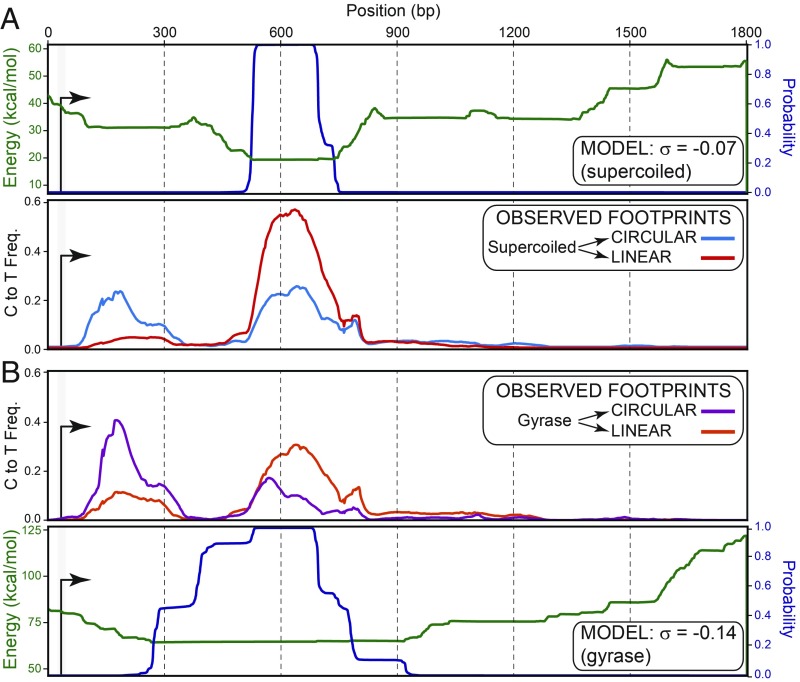
(*A*, *Upper*) Model-derived graphs for the ensemble average energy (green curve) and probability (blue curve) of R-loop formation for supercoiled pFC53 (data from Fig. 5*B*). (*A*, *Lower*) The average C-to-T conversion frequencies for R-loop peaks formed on supercoiled pFC53 before and after linearization are shown by blue and red curves, respectively. (*B*) Similar to *A*, except that the order of *Upper* and *Lower* were reversed and shown for hypernegatively supercoiled (gyrase-treated) pFC53.

Our model makes two additional predictions. First, increasing negative superhelicity should permit R-loop formation for sequences with suboptimal base-pairing energies (Fig. 2). Second, R-loops formed in less-favorable regions should require superhelicity to maintain their stability. To test this, we in vitro-transcribed supercoiled pFC53 and used SMRF-seq to map R-loops either directly on circular molecules or after linearization (*SI Appendix*, Fig. S6*A*). A prominent class of R-loops was detected on circular molecules closer to the TSS, in regions with modest or poor predicted energetic favorability (Fig. 6*A*). These footprints accounted for 33% of all R-loops detected in circular plasmids, compared with 6.2% after linearization (*SI Appendix*, Fig. S5). By contrast, footprints over the most favorable region only accounted for 56% of the total on circular molecules compared with 85% after the DNA was cut. The fraction of molecules carrying double R-loops also decreased from 4.8% when circular to 1.2% when linear (*SI Appendix*, Fig. S5). From this, we conclude that circular molecules harbored a class of suboptimal R-loops that were preferentially lost upon linearization. As a result, stable R-loops that remained after linearization mostly matched the most energetically favorable regions.

To test how increasing negative superhelicity affects the distribution and stability of R-loops on linear vs. circular molecules, we performed SMRF-seq on gyrase-treated pFC53 after in vitro transcription. When assayed on circular molecules, a majority (55%) of footprints were now observed close to the TSS (Fig. 6*B* and *SI Appendix*, Fig. S5), and only 32% of R-loops matched the most favorable region. Part of this shift toward the TSS was predicted by our model (Fig. 6*B*), but a significant fraction of R-loops was located outside of any high-favorability region. The bulk of these structures were again lost after linearization. As a result, only a minority of linear molecules carried any R-loops, even though, overall, R-loop formation was most efficient on highly negatively supercoiled molecules (Fig. 4). Similar results were observed with pFC8 (*SI Appendix*, Fig. S6). We conclude that increasing negative superhelicity allows R-loop formation over suboptimal sequences, as predicted. These observations are consistent with prior in vitro work (47). A clear trend for formation in close proximity to the TSS was also revealed, suggesting that suboptimal R-loops may form before transcription has reached more favorable downstream sequences. These results demonstrate that negative superhelicity can significantly alter the R-loop landscape and that it is essential for the stability of suboptimal structures.

## Discussion

R-loops are known to rely on favorable DNA base-pairing energetics and negative superhelicity for their formation. However, a clear biophysical understanding of the roles these two factors play, and of how they interact, is still lacking. Here, we developed an energy-based model for R-loops at equilibrium. Our approach included all sources of energy—junctions, superhelical deformations, and the conformation of the unpaired strand, as well as duplex disassociation and hybrid formation. Our theoretical model and its experimental validation provide a view of R-loop formation that has important implications for genome dynamics.

### Negative Superhelicity Is Critical for R-Loop Formation.

Our model predicts that DNA superhelicity is required to stabilize R-loops, even for energetically favorable sequences (Figs. 1 and 2). In vitro transcription assays confirmed that negative superhelicity strongly facilitates R-loop formation, as suggested (48); extra negative superhelicity further increased the reaction efficiency (Fig. 4). Relaxation of the template via linearization or nicking greatly reduced R-loop formation (Fig. 3), although R-loops still occurred, in agreement with earlier work (46, 48, 49). By contrast, R-loops did not form on the closed, relaxed templates examined here.

These effects can be understood by considering that negative supercoiling represents a high-stress, high-energy state and that R-loop formation alters the distribution of superhelicity in a way that relieves this stress. Specifically, in forming an R-loop, the two DNA strands separate, so the unpaired strand can wind around the hybrid. The changes of twist that occur due to these two effects localize negative superhelicity within the R-loop zone. In the context of a negatively superhelical domain, the rest of the domain will relax by a corresponding amount, and the superhelical energy returned by this relaxation favors the R-loop state. By contrast, in a topologically closed but relaxed domain (i.e., Top1-treated), the separation of the DNA duplex over the R-loop induces positive superhelicity elsewhere, which is energetically unfavorable, and hence impedes R-loop formation. In agreement, R-loop formation induced upon transcription of an extremely favorable (AGGAG)_28_ repeat sequence on relaxed substrates forced the DNA into a positively supercoiled state (46). For nicked and linear DNA, the excess positive supercoils induced by R-loop formation are eliminated by rapid strand rotation, so superhelicity neither impedes nor facilitates this reaction. The lack of ambient superhelicity, however, prevents energy return from DNA relaxation, and therefore R-loop formation is significantly lower on these substrates (Fig. 4).

Intriguingly, our model predicts that a high level of positive superhelicity also favors R-loops (Figs. 2 and 3). Similar behavior has been noted for strand separation (50, 51). Whether this prediction is borne out in vitro or in vivo remains to be tested. We note that positive superhelicity may hinder transcription elongation (52). It also remains unclear if the displaced ssDNA can sterically wind around the RNA:DNA hybrid enough to permit relaxation of positive supercoils. Such winding would require the single strand to stretch nearly to its physical limit, and in these conditions, the energetics might be different from those assumed in the model. Furthermore, very few genomic loci are expected to experience levels of positive superhelicity sufficient to drive R-loops. Such regions are likely limited to loci between nearby convergent highly transcribed genes or convergently advancing transcription and replication forks. Even in these rare cases, the positively supercoiled region would be located ahead of the RNA polymerase and therefore would be untranscribed; as such, there would be no complementary transcript available to form an R-loop, unless the RNA polymerase undergoes backtracking. Overall, although R-loop formation at high positive superhelical densities is possible according to our model, its occurrence and relevance to in vivo situations remains to be established.

### R-Loop Distribution Patterns Are Governed by the Interplay Between Sequence, Superhelicity, and Proximity to the TSS.

In our model, the substantial energy needed to make a pair of junctions is the main barrier to R-loop formation. This barrier can only be overcome by a combination of superhelical relaxation and favorable base-pairing energetics. Our model predicts that for moderately favorable DNA sequences, only modest levels of negative superhelicity (σ ≅ −0.02) are sufficient to stabilize R-loops. These levels occur during normal physiological processes in all organisms. In mesophilic bacteria, gyrase activity maintains genomic DNA at unconstrained superhelical densities that can reach beyond σ = −0.05 (53). In eukaryotes, DNA replication, transcription, and chromatin-remodeling transiently introduce negative superhelicity (54, 55). During transcription, DNA is negatively supercoiled to a density of σ = −0.07 in a region extending 1,500 bp behind the advancing RNA polymerase (32, 56), which is where R-loops are thought to form. Thus, the topological requirements revealed here are compatible with R-loop formation in vivo.

Our model and its experimental validation established that superhelicity, together with DNA sequence, cooperate to regulate both the positions and the stability of R-loops. Consistent with the well-known association of R-loops with GC skew in vivo and in vitro (3, 4, 16, 24), our model predicts that R-loops will tend to concentrate over the most energetically favorable sequences, ensuring maximal energy return from RNA:DNA base-pairing. These regions tend to be G-rich. However, GC skew by itself was found to be only moderately predictive of R-loop locations (Fig. 5). Single-molecule R-loop footprinting revealed that stable R-loops—i.e., those that persist after linearization of the circular molecules on which they were generated—show remarkable agreement in position with model-predicted favorable regions (Fig. 5). Thus, cotranscriptional R-loop formation in vitro, an inherently dynamic process, follows, at least in part, the energy landscape highlighted by our equilibrium model. Our model also explicitly predicts that the position of R-loop-prone regions will shift with varying levels of superhelicity. More favorable sequences require less energy return from superhelicity and can therefore transition into R-loops at lower negative superhelical densities. By contrast, R-loops formed over suboptimal sequences are expected to require more energy return from superhelicity. At its extreme, any sequence, however unfavorable, could transition into an R-loop, provided sufficient negative supercoiling exists (Figs. 2 and 3). Experimental validation showed that TSS-proximal R-loops formed over unfavorable regions significantly increased in representation when the superhelical density of the plasmid template was increased upon gyrase treatment (Fig. 6 and *SI Appendix*, Figs. S5 and S6). Thus, increased negative superhelicity drives R-loop formation over unfavorable regions, consistent with prior in vitro work (47). Finally, our model predicted that R-loops should show differential stability in the face of a loss of superhelicity. R-loops formed over favorable regions should remain more stable because base-pairing energy provides significant anchoring. By contrast, the stability of R-loops formed over less favorable regions should be strongly compromised by relaxation. These expectations were validated by measuring the distribution of R-loop footprints generated on circular molecules after linearization of the template (Fig. 6 and *SI Appendix*, Figs. S5 and S6). TSS-proximal R-loops matching poorly favorable sequences were sharply reduced after linearization. By contrast, R-loops formed over favorable regions remained mostly stable and accounted for the majority of structures postcleavage. Our work therefore demonstrates that superhelicity can dramatically affect the R-loop landscape, both in terms of their formation and stability.

Our data also confirmed a propensity for R-loops to form in proximity to the 5′ end of the nascent transcript (48, 57), even when the sequence was suboptimal and the resulting R-loops unstable. This suggests that R-loops that form first because they are closest to the TSS may benefit from a dynamic advantage during transcription. This class of unstable, dynamic R-loops may provide promoter-proximal topological relief, even if it is to the detriment of stable R-loop formation at more favorable regions located downstream.

### R-Loops as Reversible Superhelical Stress Relievers.

Before our work, the underwound state of negatively superhelical DNA was understood to favor R-loops by facilitating the ability of the nascent RNA to invade duplex DNA (1). However, superhelical duplex destabilization is confined to the AT-richest regions of a domain (58), whereas most R-loops occur in G-rich locations. Thus, supercoiling-induced strand separation may not be strictly necessary for R-loop initiation. Our energy-based equilibrium model provides an alternative, more quantitative explanation for how negative superhelicity facilitates R-loops. Both in silico and in vitro (Fig. 3), a major effect of R-loop formation is to return a negatively supercoiled DNA domain to an energetically more favorable, nearly relaxed state. Thus, negative superhelicity favors R-loops because these structures relax superhelical stresses.

It is useful to contrast R-loop-mediated relaxation with that provided by nucleosomal winding. In vitro, R-loops of lengths <150 bp (Fig. 5) efficiently relaxed 3- to 4-kb negatively supercoiled plasmids (Fig. 3). Assuming a superhelical density of σ = −0.05, these R-loops absorbed a large fraction of the 15–20 negative supercoils present on these plasmids. By comparison, a nucleosome occupying ∼146 bp of DNA absorbs one negative supercoil (59–61). Thus, R-loops are at least an order of magnitude more effective than nucleosomes at absorbing negative superhelicity. Single-molecule R-loop profiling studies have shown that genomic R-loops often extend for several hundred base pairs, and kilobase R-loops have been detected at lower frequencies (3–5). R-loops having these lengths are capable of absorbing striking amounts of negative superhelicity and therefore can act as efficient superhelical stress relievers over long domains. Importantly, the superhelicity stored in an R-loop is immediately returned to the DNA fiber upon R-loop resolution. As shown in Fig. 3*C*, the R-loop-mediated relaxation of plasmid DNA is entirely shifted back to the negatively supercoiled state by RNase H treatment.

The ability of R-loops to efficiently sequester and release negative superhelicity may be relevant for several processes. First, it will influence the landscape of non-B DNA structures in surrounding regions. Indeed, other alternative DNA structures, such as strand separations, cruciforms, and B/Z transitions, also can be superhelically driven (34–39). In effect, all of the possible DNA structural transitions, including R-loops, will compete together because the superhelical relief caused by the formation of any one structure will inhibit the formation of all others. Given their lengths, stabilities, and remarkable capacity for absorbing superhelicity, R-loops are expected to play a major role in these competitions. Second, since negative supercoiling favors strand opening, which is required for the initiation of transcription and of DNA replication (62), these processes may be strongly influenced by the formation or resolution of nearby R-loops. CpG island promoters, which serve both as active promoters and as early, efficient DNA replication origins (63), are R-loop hotspots (4). More broadly, the identification of replication “initiation zones” near gene ends (64) matches well with the known favored locations for R-loops at the beginnings and ends of genes. Finally, the sequestering or release of negative superhelicity by R-loop formation or resolution could influence the landscape of protein–DNA interactions, since proteins such as transcription factors and nucleosomes preferentially bind negatively supercoiled DNA (65, 66). Indeed, the release of negative superhelicity upon R-loop resolution may permit efficient nucleosome redeposition locally, since R-loops are most likely nucleosome-free as long as they persist (11, 67).

Finally, the observation that negative DNA superhelicity enhances the formation and stability of R-loops suggests that genomic sites where R-loops occur exist under topological tension. If so, genome-wide R-loop maps may indirectly report on local levels of superhelicity. CpG island promoters experience repeated topological strain caused by transcription initiation, including abortive transcription and nucleosome remodeling (32, 56). The observation of strong R-loop hotspots at these sites is consistent with R-loops providing a nonenzymatic method to regulate that strain. The fact that CpG island promoters across vertebrates contain R-loop-favorable sequences (4, 15) suggests that these loci may have evolved to take advantage of R-loop-mediated torsional stress relief. Interestingly, the 3′ ends of numerous genes are strong R-loop hotspots (11). These regions may therefore also experience significant topological tension, perhaps linked to transcription termination. Overall, our work suggests that R-loops can provide ways to distribute and manage topological stresses in eukaryotic genomes.

## Materials and Methods

For more information on the derivation of the equilibrium model, including the implementation of the model in the R-looper algorithm, please consult the *SI Appendix*. The *SI Appendix* also includes information regarding reagents, chemicals, and procedures related to plasmid DNA extraction and topological manipulations and in vitro transcription assays. Single-molecule R-loop footprinting assays are also described in the *SI Appendix*.

## Supplementary Material

Supplementary File
