# Supplementary material for: Interplay between DNA sequence and negative superhelicity drives R-loop structures
Source: Proc Natl Acad Sci U S A. 2019 Mar 8;116(13):6260–9. doi: 10.1073/pnas.1819476116 (PMC6442632; doi:10.1073/pnas.1819476116)
Supplement: Supplementary File [file pnas.1819476116.sapp.pdf]

Supplementary Information

Supplementary Methods

Figures S1 to S6

Supplementary References

www.pnas.org/cgi/doi/10.1073/pnas.1819476116

Supplementary Methods

Derivation of the equilibrium energy model. Consider a topological domain containing N DNA base pairs of any specified sequence. This could be either a closed circle or a topologically constrained loop. Suppose the domain is superhelically constrained to have linking difference α (i.e. when the domain is held in a planar configuration, its interstrand twist differs from the relaxed B-form value by α turns). This superhelicity is determined only by the arrangement in space of the two DNA strands in the domain. Whether or not one of these strands is bound to an RNA, and the helicity of that binding, have no effect on superhelicity. While α has the same value in all states of this domain, it may be partitioned in different ways.

Suppose for simplicity that R-loops can occur at any position in this domain, and can have any length up to the maximum allowed by the sequence. This assumes there are complementary RNAs available over the entire sequence. If not, one can restrict calculations to those portions of the domain where RNA transcripts exist. We calculate the equilibrium distribution of a population of identical molecules among all possible R-loop states (i.e. states of the domain whose superhelicity is α).

Equilibrium Statistical Mechanics The relative frequency with which a specific state s occurs at equilibrium depends on the (free) energy $G(s)$ of that state as $e^{-G(s)/RT}$. Thus, the occupancies of individual states decrease exponentially as their energies increase. According to standard statistical mechanics, the equilibrium probability of state s', which is the frequency of that state in the equilibrium distribution, is given by:

$$p(s') = \frac{e^{-G(s')/RT}}{\sum_s e^{-G(s)/RT}}. \qquad \text{(S1)}$$

Here the denominator is summed over all possible R-loop states s, including the state with no R-loop. If a parameter q has value $q(s)$ in state s, then its ensemble average value at equilibrium is

$$\bar{q} = \sum_s q(s)p(s)\,. \qquad \text{(S2)}$$

In this way one can compute equilibrium values of any parameter of interest. These include the fraction of molecules that have an R-loop, the average length of these R-loops, and the probability that any specific base pair occurs in an R-loop. To perform these calculations one must specify the (free) energies $G(s)$ associated to R-loop states.

Energetics of R-loop States. Consider a domain that contains r R-loops converting a total of m DNA:RNA hybrid base pairs. Unless r = 1, these base pairs will not all be sequential. If the domain is circular, care must be taken to include states where R-loops can span the origin.

The energy associated with this state has four terms. Two energy terms come from the cost of forming R-loops. First, the base pair energy $B(x) = b_{hyb}(x) - b_{dup}(x)$ is the (free) energy required to locally denature the DNA duplex base pair at position x, and form a DNA-RNA hybrid base pair in its place. Second, at the start and the end of each R-loop there are two junctions between the hybrid and the duplex. The free energy required to form this pair of junctions is denoted by a. (The assignment of values to all energy parameters is discussed in the next section). Thus, the energy G_r required to form the r R-loops in this state is:

$$G_r = ra + \sum_{i=1}^{m} B(i)\,. \qquad \text{(S3)}$$

Here the sum is over the m base pairs that occur in R-loops.

The last two contributions to the energy of this state derive from the repartitioning of superhelicity that occurs when R-loops are present. Dissociation of the two DNA strands changes the unstressed twist of the m base pairs involved from mA characteristic of the duplex, to zero. (Here A =1/10.5 turns/bp is the average helicity of B-form DNA). This untwisting absorbs negative superhelicity, leaving α + mA superhelical turns available to stress the rest of the domain. Finally, because single stranded DNA is flexible, the unpaired, non-template strand can wind around the DNA:RNA hybrid with helicity τ (radians per base). This leaves a residual superhelicity:

$$\alpha_r = \alpha + mA - \frac{m\tau}{2\pi}\,. \qquad \text{(S4)}$$

The free energy associated to superhelicity α is known to be quadratic (1):

$$G(\alpha_r) = \frac{1}{2}K\alpha_r^2\,. \qquad \text{(S5)}$$

The free energy associated to the helical winding of the single strand about the duplex is modeled as having a Hooke's law quadratic energy in τ:

$$G(\tau) = \frac{1}{2}mC\tau^2, \qquad \text{(S6)}$$

where C is the torsional stiffness of this winding. The total energy due to these two effects is:

$$G(\alpha_r, \tau) = \frac{1}{2}K\alpha^2 + \frac{1}{2}mC\tau^2, \qquad \text{(S7)}$$

while the two parameters α_r and τ are coupled together as:

$$\alpha_r + \frac{m\tau}{2\pi} = \alpha + mA\,. \qquad \text{(S8)}$$

Because both the superhelicity α and the base pairs participating in R-loops have been fixed for this state, the right hand side of this equation is a constant. A simple calculation shows that the minimum value of the energy in Eq. (S7) subject to the coupling constraint given in Eq. (S8) occurs when $2\pi C\tau = K\alpha_r$. So

$$\alpha_r = \frac{4\pi^2 C}{4\pi^2 C + Km}(\alpha + mA), \qquad \text{(S9)}$$

and

$$\tau = \frac{2\pi K}{4\pi^2 C + Km}\left(\alpha + mA\right). \qquad \text{(S10)}$$

Substitution of these results in Eq. (S7) shows the minimum value $G_{\min}$ of the free energy $G(\alpha_r, \tau)$ to be

$$G_{min} = \frac{2\pi^2 CK}{4\pi^2 C + Km}\left(\alpha + mA\right)^2. \qquad \text{(S11)}$$

Finally, the free energy of this state is modeled as:

$$G(s) = G_r + G_{min} = \begin{cases} \frac{1}{2}K\alpha^2 & \text{if } m = 0, \\ ar + \sum_{i=1}^{m} B(i) + \frac{2\pi^2 CK}{4\pi^2 C + Km}\left(\alpha + mA\right)^2 & \text{if } m > 0. \end{cases} \qquad \text{(S12)}$$

<u>Assignment of Parameter Values</u> The base pair energy $B(x) = b_{hyb}(x) - b_{dup}(x)$ required to locally denature a DNA duplex base pair, and form a DNA-RNA hybrid base pair in its place has been provided for all 16 nearest neighbor base pairs (2). Seven of these values are negative, meaning that for them the DNA:RNA hybrid is actually more stable than the DNA:DNA duplex. This stands in contrast to other alternative DNA structures, such as Z-form, cruciform, and

strand separation, all of which are energetically unfavorable relative to the B-form under physiological conditions.

While the junction free energy a has not been measured specifically for R-loops, it is known that both B-Z transitions and local strand separations have junction energies in the range $10 < a < 11$ (kcal/mol) (3-6). So in our initial R-loop calculations we used a = 10.5 kcal/mol. This could be an underestimate, as R-loop junctions involve interactions among three strands, whereas these other types of junctions involve only two. This high value of a makes it energetically expensive to initiate R-loops, creating an energy barrier that can only be overcome by other, more energetically favorable factors. In practice these will be a combination of favorable sequences and superhelical relaxation. It also makes it unlikely that more than one R-loop will be energetically favored and therefore statistically plausible, as the opening of a second site would be at a less favorable position and the superhelicity already would be partially or fully relaxed by the first R-loop. In light of this, in our initial calculations we only consider states with at most one R-loop. This sets r = 1 in Eqn (S11) and Eqn. (2).

The quadratic coefficient K for the energy of superhelicity has been measured to be $K = 2200RT/N$ (1). In the present calculations we use the value C = 3.6 kcal/mol for the torsional stiffness of single strand winding. This is the value measured for the strand separation transition, where twisting of two unpaired strands occurs (3). As there is only one strand twisting in an R-loop, it is possible that this value is high.

All of the parameter values used in this initial implementation were derived from the literature; none came from experiments on R-loops. In all cases the experimental buffer and environmental conditions were somewhat different from the ones in which our R-loop experiments were performed. Despite this, the present theory provides highly accurate predictions of the occurrence, locations and sizes of R-loops. In future work we hope to determine values for these parameters that are specific to R-loops, which should increase the quantitative accuracy of our model predictions.

Implementation of the energy model in the R-Looper Program. We have implemented the calculation of R-loop equilibria in a C++ program called R-looper, which is available for download at https://github.com/chedinlab/rlooper. The user inputs the sequence to be analyzed as a FASTA file, states whether or not that sequence is circular, and specifies the superhelicity. Then the computer enumerates all single R-loop states in that sequence, plus the state of no transition, with the free energy of each state determined from Eq. (1). Insertion of these values into Eq (S1) calculates the probabilities *p*(s) of all states, which gives the equilibrium distribution. Then Eq (S2) allows us to calculate the equilibrium ensemble average values of any parameters of interest. These include the probability of R-loop formation as a function of the imposed superhelicity α, the expected R-loop length as a function of α, and the transition profile (i.e. the probability of each base pair in the sequence participating in an R-loop). Specific predictions of this theory made using R-looper are presented in the body of the paper. For figures 1 and 2, calculations were performed on a 1,500 bp superhelical domain at the given superhelicities.

Plasmids and topological manipulation. Negatively supercoiled plasmid substrates were purified by cesium chloride ultracentrifugation gradients and their topology enzymatically manipulated. To create relaxed closed DNA substrates, plasmids were incubated with *E. coli* DNA topoisomerase I (New England Biolabs) for 1 hr according to the manufacturer's instructions. To produce topologically unconstrained DNA, plasmids were either subjected to a single nick immediately upstream of the promoter using the BspQI nicking enzyme or cut into two restriction fragments using ApaLI (New England Biolabs). To generate a highly negatively supercoiled population, the plasmid was treated with *E. coli* DNA gyrase (New England Biolabs) for 30 min according to the manufacturer's instructions. The resulting changes in DNA topology were verified on 1D and 2D chloroquine gels (Figure S2) prior to *in vitro* transcription. For 1D gels, plasmid DNAs were separated through 1% agarose gels with 2.5 μg/ml chloroquine for 16 hours at 110 volts in 2x TBE buffer. For 2D gels, DNAs were first separated through 0.6% agarose gels with 0.5 μg/ml chloroquine for 100 minutes at 120 volts in 2x TBE buffer. For the second dimension, we used 1% agarose gels with 2.5 μg/ml chloroquine and the gels were run for 16 hours at 110 volts in 2x TBE buffers.

***In vitro* transcription assays**. *In vitro* transcription assays were performed essentially as described (7) with T3 RNA polymerase (Promega). Reactions were stopped with addition of 10 mM EDTA and treated with RNase A (0.1 mg/ml) for 30 minutes at 37°C to degrade excess free RNA. The reaction products were then digested with Proteinase K for 30 min. For direct visualization (inset in Figure 3C), the products were separated by agarose gel electrophoresis and post-stained with ethidium bromide to visualize the topological relaxation caused by R-loop formation. To visualize potential R-loops in relaxed substrates for which further DNA relaxation

can’t be detected (e.g. relaxed, nicked and linear substrates), the products were purified by phenol/chloroform extraction and ethanol precipitation, resuspended in TE and incubated with the S9.6 antibody to supershift any RNA:DNA hybrids. The products were then separated by agarose gel electrophoresis and post stained with ethidium bromide.

SMRF-seq based R-loop footprinting after *in vitro* transcription. *In vitro* transcription was performed as described above on supercoiled DNA templates, followed by RNase A treatment, ApaLI digestion, phenol/chloroform extraction and ethanol precipitation. The DNA was then treated with sodium bisulfite using the ZymoGold bisulfite modification kit (Zymo Research) according to the manufacturer’s instructions except that treatment was performed under non-denaturing conditions at 37°C for 3 hrs. Following sample clean-up, the DNA was amplified using native primers flanking the R-loop prone region and the PCR amplicons were purified and processed directly to build PacBio sequencing libraries. Upon library validation, sequencing was performed on pooled barcoded libraries on a Pacific Biosciences RSII instrument. Following sequencing, circular consensus sequences (CCS) were created using standard PacBio pipeline enforcing a minimal 3x pass. After additional quality control and length filtering, reads were debarcoded and mapped to the reference plasmid genomes using the bisulfite-enabled Bismark mapping package (8). C to T conversion signals were then assessed using the Footloop package which will be described elsewhere and is available upon request. In brief, we used a window-based method to call peaks of signal taking into account a minimum of 15 cytosines per window and enforcing a threshold of 55% conversion and a minimum length of 30 nucleotides to call a given window as a peak. Converted cytosines that were not part of a peak were also called separately.

SUPPLEMENTARY FIGURES

Figure S1

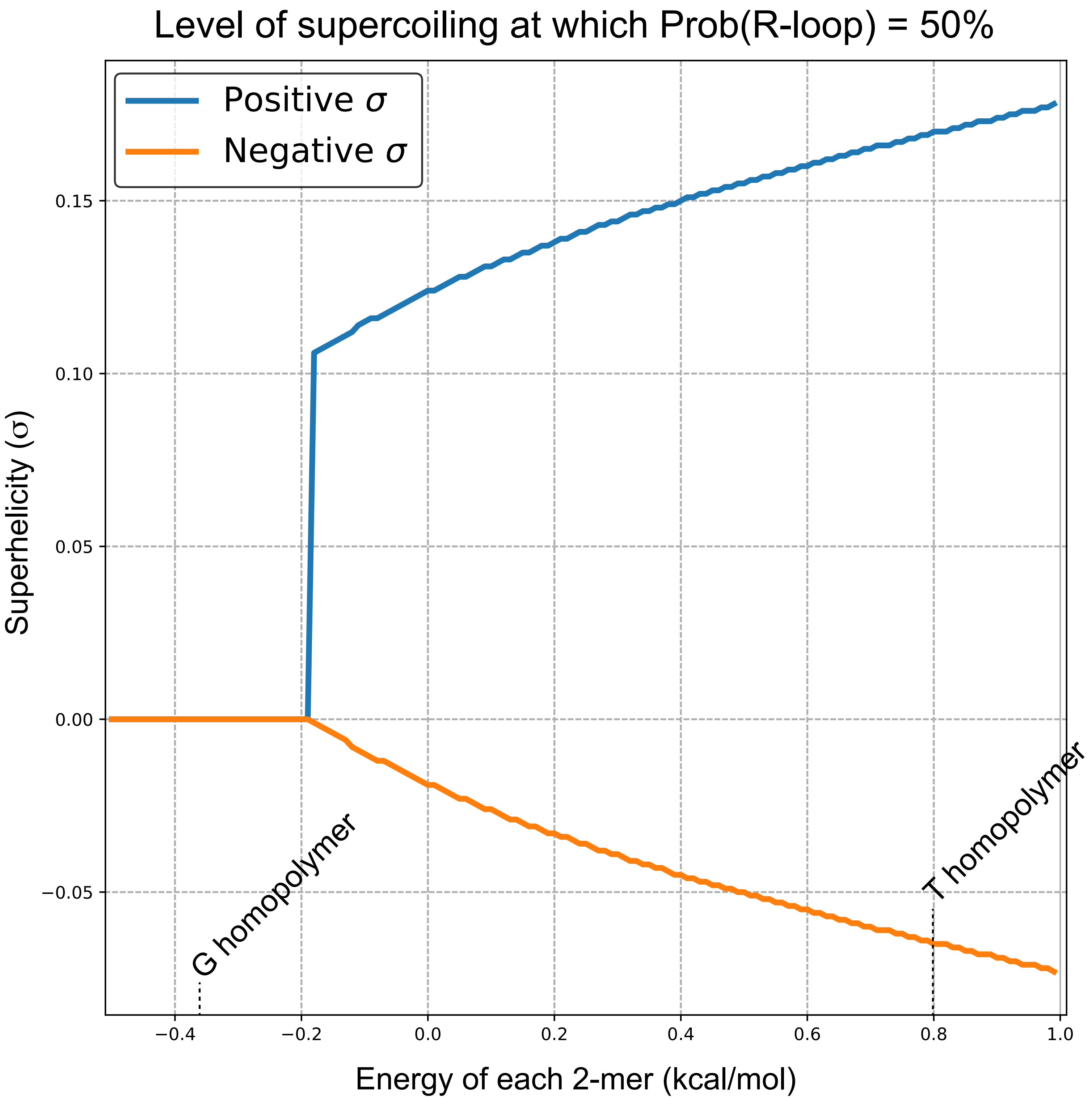

Level of supercoiling at which Prob(R-loop) = 50%
Positive σ
Negative σ
0.15
0.10
0.05
0.00
−0.05
Superhelicity (σ)
G homopolymer
T homopolymer
−0.4
−0.2
0.0
0.2
0.4
0.6
0.8
1.0
Energy of each 2-mer (kcal/mol)

Figure S1: this graph depicts the superhelicity (y-axis) required to achieve a 50% probability of R-loop formation as a function of the overall base-pair energy of the DNA sequence (x-axis). The calculation assumes that each sequence is uniform in its base-pair energy. Below -0.2 kcal/mol, the base-pairing energy is so favorable that superhelicity is not required to form R-loops. Above this value, R-loop formation in a negatively supercoiled superhelical domain requires gradually more negative superhelicity to stabilize R-loops. By contrast, R-loop formation in a positively supercoiled superhelical domain requires a large jump of positive superhelicity to stabilize R-loops. The energies associated with G and T homopolymers are shown on the x-axis to highlight the range of base-pair energies that are achievable in actual DNA sequences.

Figure S2

A.

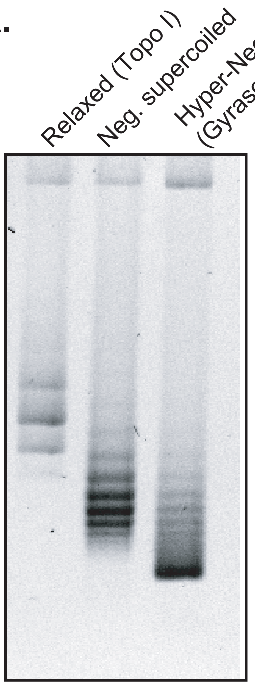

Relaxed (Topo I)
Neg. supercoiled
Hyper-Neg. (Gyrase)

B.

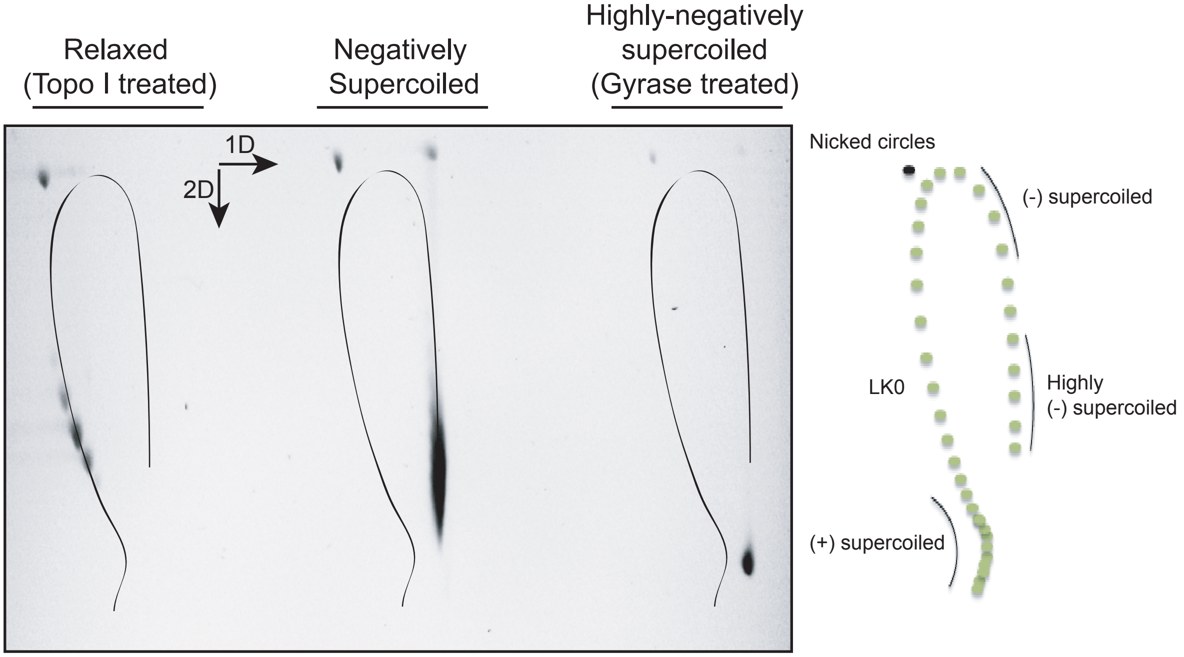

Relaxed (Topo I treated)
Negatively Supercoiled
Highly-negatively supercoiled (Gyrase treated)
1D
2D
Nicked circles
(-) supercoiled
LK0
Highly (-) supercoiled
(+) supercoiled

Figure S2: One-dimensional (A.) and two-dimensional (B.) gel electrophoresis validates that the topological manipulations used here altered the superhelical state of plasmid DNA substrates as expected. Compared to normally negatively supercoiled substrates, topoisomerase I treatment retarded the migration in 1D gels and shifted topoisomers towards relaxed circles on the left arc of 2D gels. By contrast, DNA gyrase-treated samples led to faster migration in 1D gels and topoisomers were pushed significantly further along the right arc of 2D gels. The direction of the first and second dimensions are indicated on the 2D gel. A schematic of the expected migration patterns of topoisomers under the experimental conditions used here is depicted to the right. The expected arc of migration is traced under each sample for ease of interpretation.

Figure S3

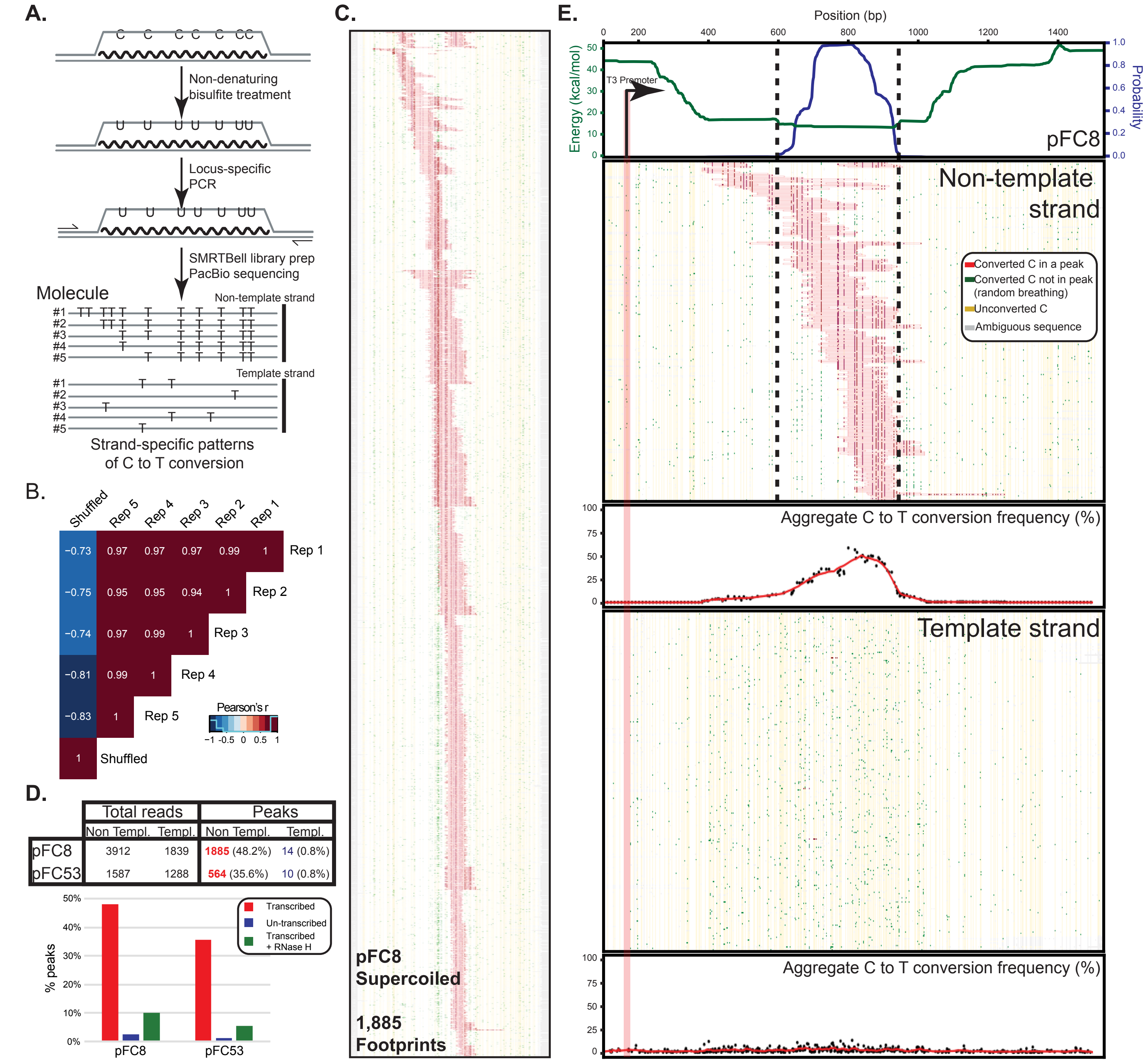
A.
Non-denaturing
bisulfite treatment
Locus-specific
PCR
SMRTBell library prep
PacBio sequencing
Molecule
Non-template strand
Template strand
#1
#2
#3
#4
#5
Strand-specific patterns
of C to T conversion
B.
Shuffled
Rep 5
Rep 4
Rep 3
Rep 2
Rep 1
−0.73 0.97 0.97 0.97 0.99 1 Rep 1
−0.75 0.95 0.95 0.94 1 Rep 2
−0.74 0.97 0.99 1 Rep 3
−0.81 0.99 1 Rep 4
−0.83 1 Rep 5
1 Shuffled
Pearson's r
−1 -0.5 0 0.5 1
C.
pFC8
Supercoiled
1,885
Footprints
D.
Total reads
Peaks
Non Templ. Templ.
Non Templ. Templ.
pFC8 3912 1839 1885 (48.2%) 14 (0.8%)
pFC53 1587 1288 564 (35.6%) 10 (0.8%)
% peaks
50%
40%
30%
20%
10%
0%
pFC8
pFC53
Transcribed
Un-transcribed
Transcribed
+ RNase H
E.
Position (bp)
0 200 400 600 800 1000 1200 1400
Energy (kcal/mol)
Probability
T3 Promoter
pFC8
Non-template
strand
Converted C in a peak
Converted C not in peak
(random breathing)
Unconverted C
Ambiguous sequence
Aggregate C to T conversion frequency (%)
Template strand
Aggregate C to T conversion frequency (%)

Figure S3: **A.** Schematic of SMRF-seq methodology. **B.** Reproducibility heatmaps for all five SMRF-seq replicates on supercoiled pFC8. Reproducibility was evaluated by comparing the positions of conversion footprints across replicates. The agreement in position was then quantified using Pearson correlation and displayed as a color heatmap of Pearson values. We computationally shuffled the positions of footprints and provide the corresponding Pearson correlation values as a null control. **C.** All 1,885 footprints collected over the pFC8 R-loop prone region (data from Figure 5A) are arranged horizontally. Each horizontal line represents one independent DNA molecule carrying an R-loop. Color codes for the heatmap are shown on panel E and explained below. **D.** Summary of SMRF-seq data. Reads and peaks are broken between the Template (T) and Non-Template (NT) strands. SMRF-seq data obtained without transcription and after transcription and RNase H treatment are shown. A graph of the percent peaks observed in all three sample types is shown below. **E.** (Top) Ensemble average R-loop energy profile and probability over the pFC8 amplicon; the position of the T3 promoter is indicated. The dotted lines extending down to the next panel highlight the region of significant predicted R-loop probability. The second panel displays individual R-loop footprints measured on the non-template (displaced) strand by SMRF-seq after transcription of pFC8 in a supercoiled state. Each horizontal line corresponds to an individual DNA molecule. 250 independent molecules were randomly sub-sampled from a total of 1,885 R-loops for better visualization. Long contiguous stretches of C to T conversion indicative of the presence of an R-loop are highlighted in red. Cytosines that were converted to thymine but are not part of a contiguous stretch are indicated in green. Unconverted cytosines are indicated in orange. Ambiguous sequence calls are colored in grey. The third panel displays the aggregate C to T conversion frequency measured over R-loop footprints for all R-loop-carrying molecules. The fourth panel displays typical C to T conversion patterns observed on the template DNA strand. 250 independent molecules were randomly sub-sampled from a total of 1839 molecules for better visualization; conversion reflects mostly random DNA breathing. The bottom panel depicts the aggregate C to T conversion frequency for the template DNA strand.

Figure S4

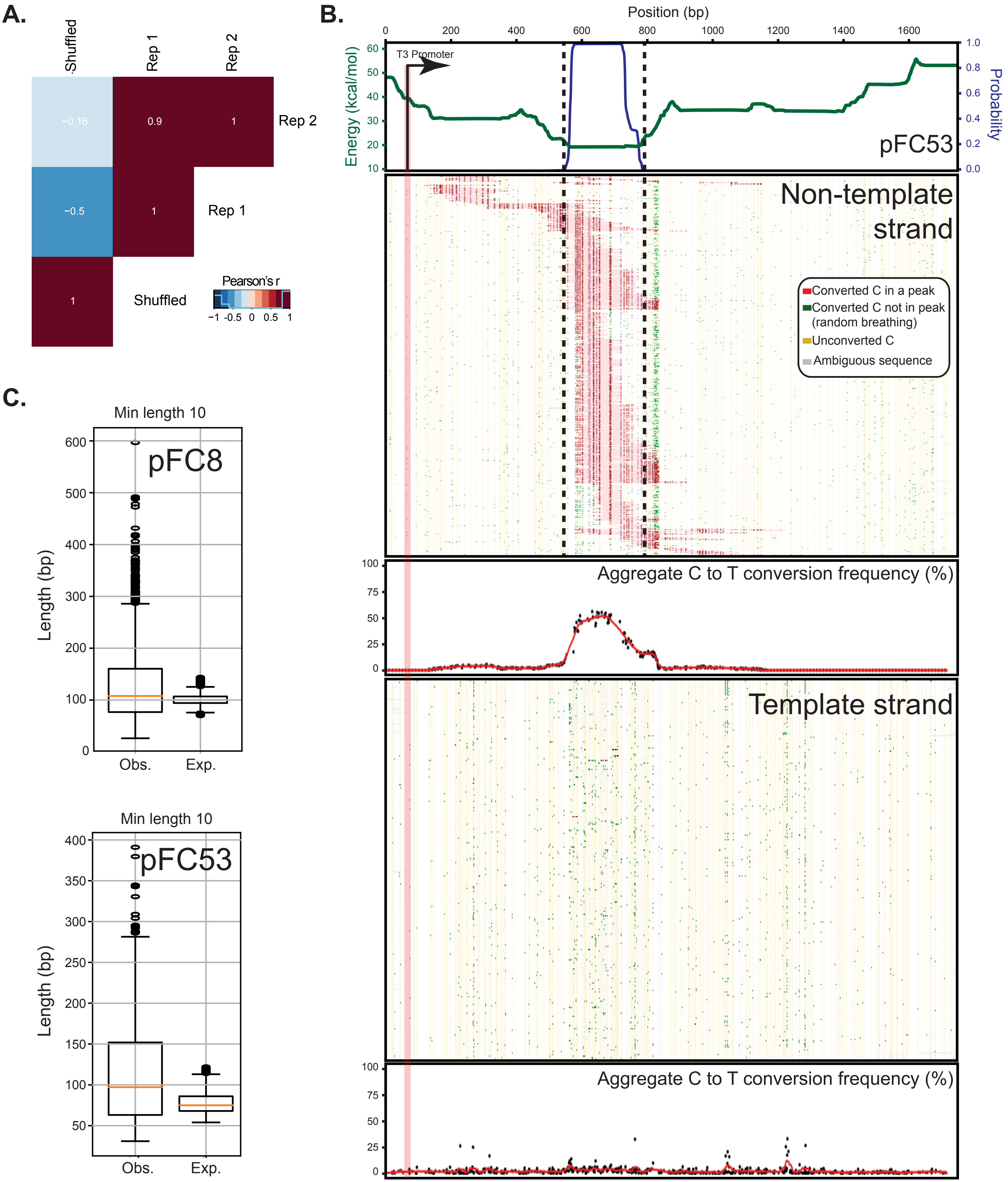
A.
Shuffled
Rep 1
Rep 2
-0.16
0.9
1
Rep 2
-0.5
1
Rep 1
1
Shuffled
Pearson's r
−1 -0.5 0 0.5 1
B.
Position (bp)
0 200 400 600 800 1000 1200 1400 1600
Energy (kcal/mol)
Probability
T3 Promoter
pFC53
Non-template strand
Converted C in a peak
Converted C not in peak (random breathing)
Unconverted C
Ambiguous sequence
Aggregate C to T conversion frequency (%)
Template strand
Aggregate C to T conversion frequency (%)
C.
Min length 10
pFC8
Length (bp)
Obs.
Exp.
Min length 10
pFC53
Length (bp)
Obs.
Exp.

Figure S4: **A.** Reproducibility heatmap for two SMRF-seq replicates on supercoiled pFC53. Visualization is as described in Figure S3A. **B.** (Top) Ensemble average R-loop energy profile and probability over a ~1,700 bp amplicon corresponding to the pFC53 R-loop prone region (data from Figure 5B). The position of the T3 promoter is indicated. The dotted lines extending down to the next panel highlight the region of significant predicted R-loop probability. The second panel displays individual R-loop footprints measured on the non-template (displaced) strand by SMRF-seq after transcription of pFC53 in a supercoiled state. Each horizontal line corresponds to an individual DNA molecule. All available footprints are shown (n = 564). Color scheme is as described for Figure S3E. The fourth panel displays typical patterns of C to T conversion observed on the template strand. No footprints can be observed and conversion is mostly random. 250 independent molecules were randomly sub-sampled from a total of 1,288 molecules for better visualization. The bottom panel depicts the aggregate C to T conversion frequency for the template DNA strand. **C.** Boxplots of observed (left) and predicted (right) R-loop lengths for pFC8 (top) and pFC53 (bottom). The minimal lengths for experimentally derived R-loops was arbitrarily cutoff at 10 bp.

Figure S5

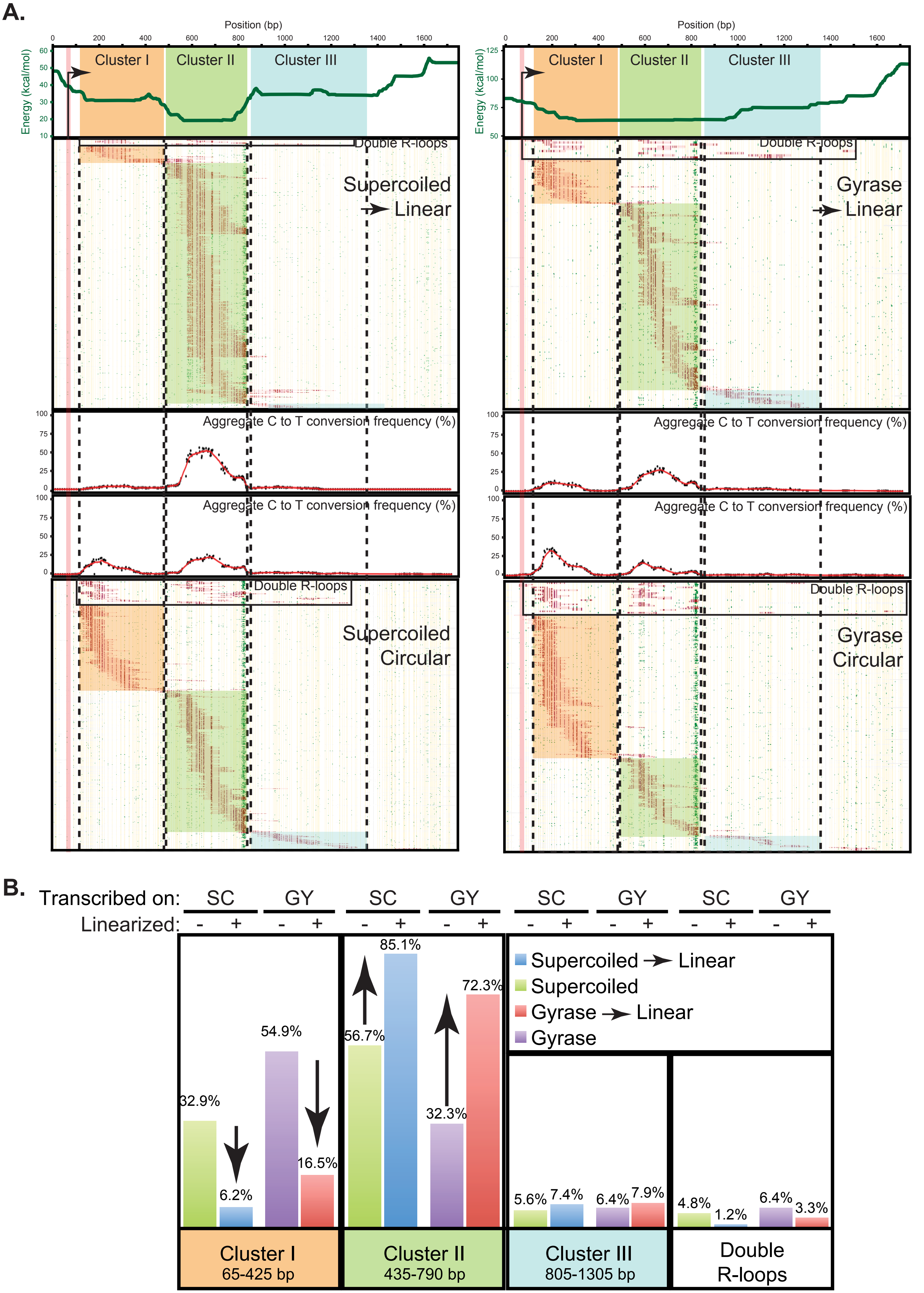
A.
Position (bp)
Energy (kcal/mol)
Cluster I
Cluster II
Cluster III
Double R-loops
Supercoiled → Linear
Gyrase → Linear
Aggregate C to T conversion frequency (%)
Supercoiled Circular
Gyrase Circular
B.
Transcribed on: SC GY SC GY SC GY SC GY
Linearized: - + - + - + - + - + - + - + - +
32.9%
6.2%
54.9%
16.5%
56.7%
85.1%
32.3%
72.3%
5.6%
7.4%
6.4%
7.9%
4.8%
1.2%
6.4%
3.3%
Supercoiled → Linear
Supercoiled
Gyrase → Linear
Gyrase
Cluster I 65-425 bp
Cluster II 435-790 bp
Cluster III 805-1305 bp
Double R-loops

Figure S5: **A**. Left. Distribution of R-loop footprints generated on supercoiled pFC53 when assayed on linear (top) versus circular (bottom) molecules. The footprints were broken down into three adjacent color-coded clusters matching to distinct sequences over the *Airn* sequence. The top graphs reprises the energetic favorability of R-loops over the amplicon as shown in Figure 5B. The middle graphs depict the average C to T conversion frequencies observed over R-loop peaks in each sample. Right. Same as left, except the data was now collected for the highly negatively supercoiled pFC53 after DNA gyrase treatment. **B**. Quantification of the distribution of R-loops along clusters. The proportion of the total distribution represented by each cluster is graphed for all three clusters and for molecules with two R-loops (located at the top of the footprint pile up in each screenshot from panel A above). The state of the DNA when transcribed (supercoiled, SC and gyrase-treated, GY) and when assayed by SMRF-seq (plus and minus linearization) is indicated above the graph and color codes are indicated in the inset.

Figure S6

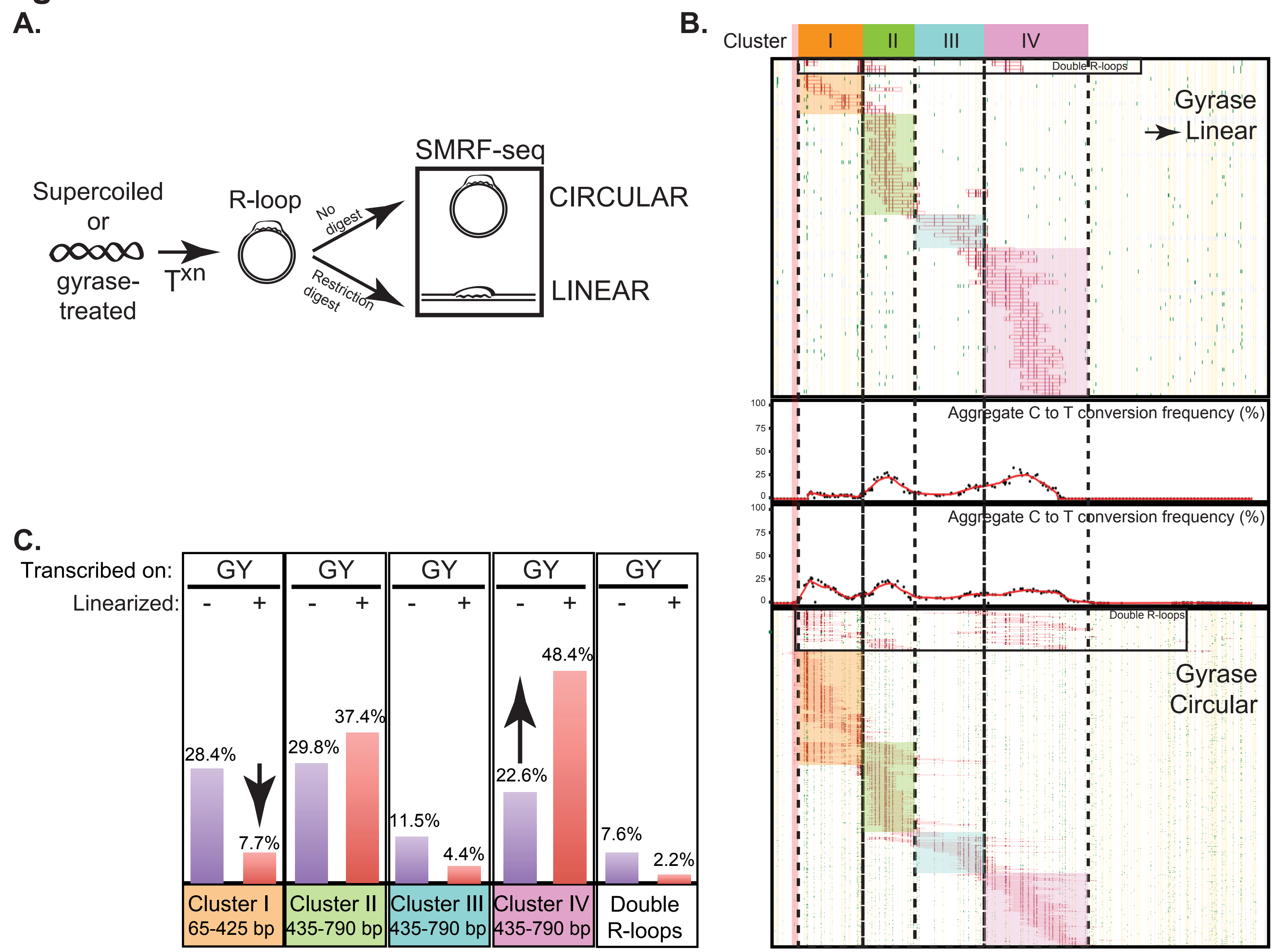
A.
Supercoiled
or
gyrase-
treated
Txn
R-loop
No digest
Restriction digest
SMRF-seq
CIRCULAR
LINEAR
B.
Cluster
I
II
III
IV
Double R-loops
Gyrase
Linear
Aggregate C to T conversion frequency (%)
100
75
50
25
0
Aggregate C to T conversion frequency (%)
Double R-loops
Gyrase
Circular
C.
Transcribed on:
Linearized:
GY
-
+
28.4%
7.7%
29.8%
37.4%
11.5%
4.4%
22.6%
48.4%
7.6%
2.2%
Cluster I
65-425 bp
Cluster II
435-790 bp
Cluster III
435-790 bp
Cluster IV
435-790 bp
Double
R-loops

Figure S6: **A.** Schematic description of the experimental scheme. R-loop distribution was measured with or without linearization after *in vitro* transcription of circular supercoiled templates. **B** and **C**. Same as Figure S5A and B except the data was obtained from highly negatively supercoiled (DNA gyrase-treated) pFC8.

Supplementary References

1. Pulleyblank DE, Shure M, Tang D, Vinograd J, & Vosberg HP (1975) Action of nicking-closing enzyme on supercoiled and nonsupercoiled closed circular DNA: formation of a Boltzmann distribution of topological isomers. *Proc Natl Acad Sci U S A* 72(11):4280-4284.
2. Huppert JL (2008) Thermodynamic prediction of RNA-DNA duplex-forming regions in the human genome. *Mol Biosyst* 4(6):686-691.
3. Bauer WR & Benham CJ (1993) The free energy, enthalpy and entropy of native and of partially denatured closed circular DNA. *J Mol Biol* 234(4):1184-1196.
4. Benham CJ (1987) Energetics of superhelicity and of B-Z transitions in superhelical DNA. *Cell Biophys* 10(3):193-204.
5. Peck LJ & Wang JC (1983) Energetics of B-to-Z transition in DNA. *Proc Natl Acad Sci U S A* 80(20):6206-6210.
6. Zhabinskaya D & Benham CJ (2011) Theoretical analysis of the stress induced B-Z transition in superhelical DNA. *PLoS Comput Biol* 7(1):e1001051.
7. Ginno PA, Lott PL, Christensen HC, Korf I, & Chedin F (2012) R-loop formation is a distinctive characteristic of unmethylated human CpG island promoters. *Molecular cell* 45(6):814-825.
8. Krueger F & Andrews SR (2011) Bismark: a flexible aligner and methylation caller for Bisulfite-Seq applications. *Bioinformatics* 27(11):1571-1572.
